# Olfactomedin-3 Enhances Seizure Activity by Interacting With AMPA Receptors in Epilepsy Models

**DOI:** 10.3389/fcell.2020.00722

**Published:** 2020-08-11

**Authors:** Shirong Tang, Tiancheng Wang, Xiaogang Zhang, Yi Guo, Ping Xu, Junwei Zeng, Zhong Luo, Dongxu Li, Yongsu Zheng, Yuemei Luo, Changyin Yu, Zucai Xu

**Affiliations:** ^1^Department of Neurology, The Affiliated Hospital of Zunyi Medical University, Zunyi, China; ^2^Department of Neurology, The Thirteenth People’s Hospital of Chongqing, Chongqing, China; ^3^Department of Neurology, Lanzhou University Second Hospital, Lanzhou, China; ^4^Department of Neurology, Chongqing General Hospital, University of Chinese Academy of Sciences, Chongqing, China; ^5^Department of Neurology, The First Affiliated Hospital of Chongqing Medical University, Chongqing, China; ^6^Department of Physiology, Zunyi Medical University, Zunyi, China; ^7^Key Laboratory of Brain Science, Zunyi Medical University, Zunyi, China

**Keywords:** epilepsy, GluA1, GluA2, OLFM3, epilepsy model

## Abstract

**Background:** OLFM3 (olfactomedin-3) is a member of the olfactomedin domain family, which has been found to stimulate the formation and adhesion of tight cell connections and to regulate cytoskeleton formation and cell migration. Differences in the gene coding for OLFM3 have been found between patients with epilepsy and controls. However, the exact role of OLFM3 in epilepsy has not been thoroughly investigated.

**Methods:** Biochemical methods were used to assess OLFM3 expression and localization in the cortex of patients with temporal lobe epilepsy and in the hippocampus and cortex of epileptic mice. Electrophysiological recordings were used to measure the role of OLFM3 in regulating hippocampal excitability in a model of magnesium-free-induced seizure *in vitro*. Behavioral experiments were performed in a pentylenetetrazol (PTZ)-induced seizure model, and electroencephalograms (EEGs) were recorded in the chronic phase of the kainic acid (KA)-induced epilepsy model *in vivo*. OLFM3 and its interaction with AMPAR (α-amino-3-hydroxy-5-methyl-4-isoxazole-propionic acid receptor) subunits were analyzed by co-immunoprecipitation.

**Results:** The expression of OLFM3 was increased in the cortex of patients with temporal lobe epilepsy and in the hippocampus and cortex of epileptic mice compared with controls. Interestingly, lentivirus-mediated overexpression of OLFM3 in the hippocampus increased the susceptibility of mice to PTZ-induced seizures, and OLFM3 knockdown had the opposite effect. OLFM3 affected AMPAR currents in a brain-slice model of epileptiform activity induced by Mg2+-free medium. We found that OLFM3 co-immunoprecipitation with GluA1 and GluA2. Furthermore, downregulation or overexpression of OLFM3 in the hippocampus affected the membrane expression of GluA1 and GluA2 in epileptic mice.

**Conclusion:** These findings reveal that OLFM3 may enhance seizure activity by interacting with GluA1 and GluA2, potentially indicating a molecular mechanism for new therapeutic strategies.

## Introduction

Epilepsy is a common chronic neurological disease, and temporal lobe epilepsy (TLE) is one of the most common types of epilepsy in adult patients (Hauser et al., 1993; Jobst and Cascino, 2015). At present, there are approximately 70 million people worldwide suffering from epilepsy, most of whom are from low- and middle-income countries (Espinosa-Jovel et al., 2018; Hafele et al., 2018). Recurrent epileptic seizures seriously affect the physical and mental health of patients (Olesen et al., 2012; Wiglusz et al., 2018) and increase the public health burden on patients, family members and society. In recent years, the public has become increasingly concerned about epilepsy (Novotna and Rektor, 2017). The specific occurrence of epilepsy and the molecular mechanisms of its development are still not completely clear (Perucca and Perucca, 2019; Wang and Chen, 2019).

OLFM3 (olfactomedin-3) is a member of the olfactomedin domain family (OLFM) (Pronker et al., 2015). Currently, 13 members of this family have been identified in mammals, while there are only 5 in humans: OLFM1, OLFM2, OLFM3, OLFM4, and MYOC (Shi et al., 2016; Koch et al., 2017). OLFM members are a group of mediators that interact with extracellular proteins, playing an important role in nervous system growth and development, cell adhesion, and cell cycle regulation (Han and Kursula, 2015). OLFM3 is expressed in the retina and brain and plays an important role in the normal development of eyes (Nakaya et al., 2013; Sultana et al., 2014). In recent years, OLFM3 has been found to be a neuronal expression protein that can stimulate the formation and adhesion of tight cell connections and regulate cytoskeleton formation and cell migration (Anholt, 2014). The AMPAR (α-amino-3-hydroxy-5-methyl-4-isoxazole-propionic acid receptor) is an ionic glutamate receptor consisting of four subunits of GluA1, GluA2, GluA3, and GluA4 (Mayer, 2016). In adult brains, GluA1-GluA2 and GluA2-GluA3 are dominant, and the role of GluA1-GluA2 is particularly important (Mayer, 2016; Gupta, 2018; Liu et al., 2018), mediating rapid excitatory synaptic transmission (Herguedas et al., 2019). The concentration of the AMPA receptor subunits GluA1 and GluA2 has been shown to be significantly reduced in hippocampal synaptosomes in the postsynaptic density of a kainic acid (KA)-based rat model of chronic TLE. These changes in synaptic AMPAR subunits may contribute to further aggravation of the excitotoxic vulnerability of neurons and have significant implications for hippocampal cognition (Egbenya et al., 2018). When a stimulus reaches the threshold, the type 1 membrane vesicular glutamate transporter (vesicular glutamate transporter 1, vGlut1) transports vesicles to the presynaptic site to stimulate the release of the excitatory neurotransmitter glutamate (Yu et al., 2018). Postsynaptic density 95 (PSD-95) plays an important role in anchoring postsynaptic AMPARs through the skeleton proteins in the postsynaptic membrane. Finally, glutamic acid interacts with AMPARs and has a fast excitatory synaptic transmission effect (Matt et al., 2018).

In recent years, differences in the gene encoding OLFM3 have been found between patients with epilepsy and controls (Heinzen et al., 2010). In addition, proteomics and mass spectrometry have revealed that OLFM3 may be involved in AMPAR complex formation (Schwenk et al., 2012; Shanks et al., 2012). Previous studies have confirmed that the AMPAR complex plays a crucial role in the occurrence and development of epilepsy (Mayer, 2016; Lorgen et al., 2017). However, how OLFM3 participates in the formation of the AMPAR complex and whether it is involved in the occurrence of epilepsy have not been studied. Therefore, we investigated whether OLFM3 interacts with AMPARs and participates in the pathogenesis of epilepsy.

## Materials and Methods

### Human Samples

In our study, all brain tissue samples were randomly selected from our brain tissue bank (Yang et al., 2018). The tissues were collected from 24 patients [13 males and 11 females; mean age 34.54 ± 1.89 years (18–53 years); mean disease course 11.33 ± 5.47 years (4–25 years)] who had been diagnosed with TLE and had subsequently undergone surgical resection. Each presurgical assessment included a detailed history and neurological examination to ensure that these patients were suitable candidates for surgery. All patients were unresponsive to three or more antiepileptic drugs (AEDs). Twelve histologically normal samples were randomly collected for use as a control group; these tissues were collected from 6 males and 6 females, with a mean age of 32.92 ± 2.20 years (range, 23–50 years). The control samples were obtained from patients who had undergone cranial surgery due to increased intracranial pressure caused by head trauma and who had no history of epilepsy, had no apparent signs of central nervous system disease and had not been exposed to AEDs. In addition, no significant differences in gender or age were observed between the patients and the control group. The clinical features of the patients with TLE and the controls are summarized in Supplementary Tables S1, S2, respectively. Given practical and ethical considerations, we were unable to obtain normal hippocampal specimens from control patients. Therefore, we only studied the (neo)cortex specimens from patients.

All clinical samples were collected after being obtained from the patients in the operating room. The samples were immediately frozen in liquid nitrogen and stored at −80°C until use for Western blotting to survey the difference between the TLE and control groups. The remaining samples were sectioned at a thickness of 10 μm and stored at −20°C for immunofluorescence analysis.

### Mouse Models of Epilepsy

Specific-pathogen-free (SPF) male C57BL/6 mice (20–24 g, 8–10 weeks old) were used for the study. The mice were obtained from the Experimental Animal Center of Chongqing Medical University and were randomly divided into a normal control group and an experimental group. All animals were housed in cages under standard conditions (12-h/12-h light/dark cycles at 23 ± 1°C, with access to food and water *ad libitum*). The pentylenetetrazol (PTZ) kindling model, the classical model for the study of epileptic susceptibility and drug screening, was generated according to the methods described in a previous study (Zhu et al., 2016). Mice were intraperitoneally injected with PTZ (35 mg/kg, Sigma-Aldrich, St. Louis, MO, United States) once every other day for a total of 15 injections (from day 1 to day 30). After each injection, all the mice were immediately observed for 30 min. The evoked behavioral seizures were rated based on Racine’s standard scale (1972). Mice with at least three consecutive seizures scoring 4 or 5 were considered successfully established kindling models. To prevent false positives in one model experiment, we used another classical model for further verification. The KA-induced chronic epilepsy model, a classical model used for research on epilepsy mechanisms, was generated as previously studied (Sada et al., 2015b; Moghbelinejad et al., 2016). Briefly, after the mice were deeply anesthetized, stereotaxic injections were performed into the dorsal blade of the CA1 area at the following coordinates with respect to the bregma [anteroposterior (AP), -1.6 mm; mediolateral (ML), -1.5 mm; dorsoventral (DV), -1.5 mm]. Mice were unilaterally injected with 1.0 nmol of KA (Sigma-Aldrich Co., St. Louis, MO, United States) in 50 nl saline, and 50 nl of saline was injected into the hippocampus in the control group. Once the mice were awake, we monitored them for stage 3–5 status epilepticus (SE) on the Racine scale (Zheng et al., 2017), and only mice with stage 3–5 SE were included in the subsequent behavioral monitoring tests. Mortality rates for PTZ- and KA-induced mouse models of epilepsy were 10–20% and 5%, respectively.

### Lentiviral Vector Injections

All recombinant lentiviral products used in this study were designed and synthesized by Hanbio Biotechnological Co., Ltd. (Shanghai, China), and the lentiviral titre was calculated as 3 × 10^8^ TU/ml. Lentiviral vectors (pHBLV-U6-Scramble-ZsGreen-Puro) containing OLFM3-short hairpin RNA (shRNA; CACTTAACAGGAGCCAAAGTGTATT) and a transgene encoding green fluorescent protein (GFP) (OLFM3-shRNA) were injected into the mouse hippocampus to locally knock down OLFM3, and lentiviral vectors (pHBLV-CMVIE-ZsGreen-Puro) encoding an amplified sequence of OLFM3 (NM_153458.3, OLFM3-LV) and GFP were injected into the mouse hippocampus to locally overexpress OLFM3. Moreover, identical lentiviral vectors containing either a non-sense control shRNA and GFP (Con-sh) or only GFP (Con-LV) were used as negative controls for the knockdown and overexpression experiments, respectively.

Intrahippocampal injection of lentiviral vectors was performed as in previous studies (Jagirdar et al., 2016; Zhang et al., 2016). Briefly, the mice were deeply anesthetized and then positioned in a stereotaxic frame (Stoelting Co., Ltd., United States). Three microliters of lentiviral particles were bilaterally injected into the dorsal blade of the CA1 area through a glass pipette (0.2 μl/min). The mice were randomly divided into the following 4 groups: OLFM3-LV, Con-LV, OLFM3-shRNA and Con-sh. Successful lentivirus infection was detected at 14, 30, and 45 days after injection by Western blotting (Supplementary Figure S2).

### Behavioral Analysis

Two weeks after intrahippocampal injections of lentiviral vectors in mice, the epilepsy models were induced as described above, and the seizures were analyzed by two researchers who were blinded to the treatment conditions. For behavioral analysis in the PTZ kindling epileptic model, evoked seizures were assessed after PTZ injection according to Racine’s standard scale. Moreover, video monitoring was used to observe spontaneous recurrent seizures (SRSs) for 1 month, and the latency and frequency of SRSs of stages 3–5 (Racine’s standard scale) were recorded in the KA-induced epilepsy model. Seizure times of 24 h/day were measured for 21 consecutive days, beginning on the 14th day after SE. Mice were sacrificed on the 35th day after SE, and the neocortex and hippocampus were collected for the following experiments.

For immunofluorescence analysis, the brain tissues were fixed with 4% paraformaldehyde and then successively incubated in 20% and 30% sucrose in PBS for 24 h each. The tissues were then sectioned at 10 μm and stored at −20°C for later experimentation. For Western blotting, the neocortex and hippocampus were immediately frozen in liquid nitrogen and then stored at −80°C for later use.

### *In vivo* Multichannel Electroencephalogram (EEG) Recordings

The local field potential recording was conducted to prevent false positives in the behavior experiment. Following anesthesia, we implanted a multichannel microwire array into the hippocampus of each mouse and performed multichannel EEG recordings as previously described (Sada et al., 2015a). EEGs were recorded *in vivo* from mice in the chronic phase of the KA-induced epilepsy model using an OmniPlex D Neural Data Acquisition System (Plexon, Dallas, TX, United States). An electrophysiological seizure was defined as a seizure with a high frequency (>5 Hz), a high amplitude (>2 times the baseline), and a duration longer than 5 s (Zheng et al., 2017).

### Immunofluorescence Staining

Immunofluorescence staining was conducted as described previously (Fachim et al., 2016; Zhang et al., 2017). The following primary antibodies were used: rabbit anti-OLFM3 (1:50, Proteintech, Wuhan, China), mouse anti-glial fibrillary acidic protein (GFAP) (1:50, Boster Bioengineering, Wuhan, China), guinea pig anti-microtubule-associated protein 2 (MAP2) (1:200, Sysy, Göttingen, Germany), guinea pig anti-vGlut1(1:200, Sysy, Göttingen, Germany), mouse anti-PSD95 (1:100, Abcam, United States), mouse anti-GluA1 (1:50, Santa Cruz Biotechnology, United States), mouse anti-GluA2 (1:100, Abcam, United States), Alexa Fluor 488-conjugated goat anti-rabbit IgG antibody (1:50, Zhongshan Golden Bridge, Inc., Beijing, China), Alexa Fluor 594-conjugated goat anti-mouse IgG antibody (1:200, Zhongshan Golden Bridge Inc., Beijing, China), and Alexa Fluor 633-conjugated goat anti-guinea pig IgG antibody (1:50, Abcam, United States). Finally, the samples were treated with 4’,6-diamidino-2-phenylindole dihydrochloride (DAPI; Sigma, St. Louis, MO, United States) for 10 min to identify the nuclei. Immunofluorescently labeled sections were imaged using a laser scanning confocal microscope (Leica Microsystems, Wetzlar, Germany) and an Olympus IX 70 inverted microscope (Olympus America, Melville, NY, United States).

### Western Blotting and Co-immunoprecipitation

#### Western Blotting

Tissue samples were collected from humans and mice for Western blotting analysis according to a previously described procedure (Zhang et al., 2016). Briefly, total protein and membrane proteins were extracted with commercial extraction kits (from Beyotime Institute of Biotechnology, Shanghai, China, and Thermo Fisher Scientific Corporation, United States, respectively) according to the manufacturers’ instructions. SDS-PAGE gels were used to separate total protein lysates, which were electrophoretically transferred to polyvinylidene fluoride (PVDF) membranes (Millipore Corporation, United States). The PVDF membranes were incubated with 5% skim milk for 1 h at 37°C to block non-specific binding. Later, the membranes were incubated overnight at 4°C with the following primary antibodies: rabbit anti-OLFM3 (1:500, Proteintech, Wuhan, China), mouse anti-GluA1 (1:200, Santa Cruz Biotechnology, United States), mouse anti-GluA2 (1:1,000, Abcam, United States), rabbit anti-GluA3 (1:1,000, Cell Signaling Technology, MA, United States), rabbit anti-GluA4 (1:500, Proteintech, Wuhan, China), mouse anti-PSD95 (1:1,000, Abcam, United States), rabbit anti-GAPDH (1:3,000, Proteintech, Wuhan, China) and rabbit anti-Na-K-ATPase (ATP1A1, 1:500, Proteintech, Wuhan, China). The secondary antibodies were incubated with the PVDF membranes for 1 h at 37°C on the next day. Enhanced chemiluminescence (ECL) reagent (Thermo, Marina, CA, United States) and a Fusion FX5 image analysis system (Vilber Lourmat Sté, Marne-la-Vallée, France) were used to visualize the bands. Finally, Quantity One software (Bio-Rad, CA, United States) was used to quantify the resulting optical density (OD) values, which were normalized to GAPDH or ATP1A1 expression.

#### Co-immunoprecipitation

Protein extracts from mouse hippocampal tissues were homogenized and then mixed with immunoprecipitation (IP) lysis buffer. Equal amounts of protein were incubated with 2 μl of rabbit IgG (Abcam, Cambridge, MA, United States) as a polyclonal isotype control, 4 μl of OLFM3, 2 μl of GluA1, 2 μl of GluA2, 2 μl of GluA3, 2 μl of GluA4, or 2 μl of PSD95 antibodies for 4 h at 4°C; then, Protein A/G agarose beads (20 μl; Santa Cruz Biotechnology, Dallas, TX, United States) were incubated with the samples overnight at 4°C. The protein-bead complexes were washed 5 times and pelleted by centrifugation. Next, the supernatants were mixed with 1 × loading buffer and heated for Western blotting, which was conducted with the same antibodies as mentioned above.

### Electrophysiology

The hippocampal slices were prepared, and whole-cell patch-clamp recordings and electrophysiological analysis were conducted as described in a previous publication (Mouro et al., 2018). First, the mice were anesthetized 14 days after intrahippocampal lentiviral injection. Glass electrodes (3–5 MΩ) were placed in the CA1 cell layer of brain slices perfused with artificial cerebral spinal fluid (ACSF; 124 mM NaCl, 3 mM KCl, 1.23 mM NaH_2_PO_4_, 2 mM MgCl_2_, 2 mM CaCl_2_, 26 mM NaHCO_3_, 10 mM glucose, and pH 7.4) to record cellular electrophysiological changes. Spontaneous epilepsy was induced using Mg^2+^-free ACSF. A whole-cell current clamp was used to record the action potentials (APs) in the CA1 region to detect cellular excitability as previously reported. A whole-cell voltage-clamp recording technique was used to record miniature excitatory postsynaptic currents (mEPSCs). The mEPSCs were recorded at −70 mV, and the brain slices were bathed with ACSF containing 1 μM tetrodotoxin (TTX) and 10 μM bicuculline. To evaluate AMPAR-mediated EPSCs, we generated evoked currents using a 400-s pulse at a rate of 0.1 Hz (intensity, 50 to 200 A) delivered by a stimulation isolation unit using an S48 pulse generator (AstroMed). A bipolar stimulating electrode was positioned in the Schaffer collaterals. The evoked currents were measured in the presence of 100 M PTX and collected at two holding potentials. At -70 mV, with application of the N-methyl-D-aspartate receptor (NMDAR)-selective antagonist D-APV (50 M), the peak amplitude of the evoked EPSCs was identified as the AMPAR-mediated current. To record the paired pulse ratio (PPR) of the AMPA-EPSCs, a bipolar stimulating electrode was positioned in the Schaffer collaterals. EPSCs were evoked at a holding potential of -70 mV in the presence of 100 M PTX and 50 M D-APV. The intervals of paired stimulations were set at 25, 50, and 100 ms. The values of the ratios were defined as [p2/p1], where p1 and p2 are the amplitudes of the EPSCs evoked by the first and second pulses, respectively (Beaudoin et al., 2018; Wang et al., 2018). All recordings were monitored with a Multiclamp 700B amplifier (Axon, United States), digitized at 10 kHz and filtered at 2 kHz. Data were collected after the currents had been stable without rundown for at least 5 min (5 neurons from 5 mice per group). Mini Analysis 6.0.1 and pClamp 9.2 software (Axon, United States) were used to analyse the recording data.

### Statistical Analysis

After verifying that the experimental data exhibited normal distributions and equal variances, we analyzed the data in SPSS 18.0 (SPSS, Inc., Chicago, IL, United States) using the Student’s *t*-test to evaluate differences between two groups and one-way ANOVA to evaluate differences among more than two groups. Each experiment was repeated three times. The χ^2^ test was used to compare gender-specific differences between patients with TLE and controls. *P* < 0.05 and *P* < 0.01 indicate statistically significant differences. These data were presented as the means ± standard error of the mean (SEM) and were analyzed using GraphPad Prism software (GraphPad Software, San Diego, CA, United States).

## Results

### Expression of OLFM3 in Patients With TLE and in Mouse Epilepsy Models

**FIGURE 1:**
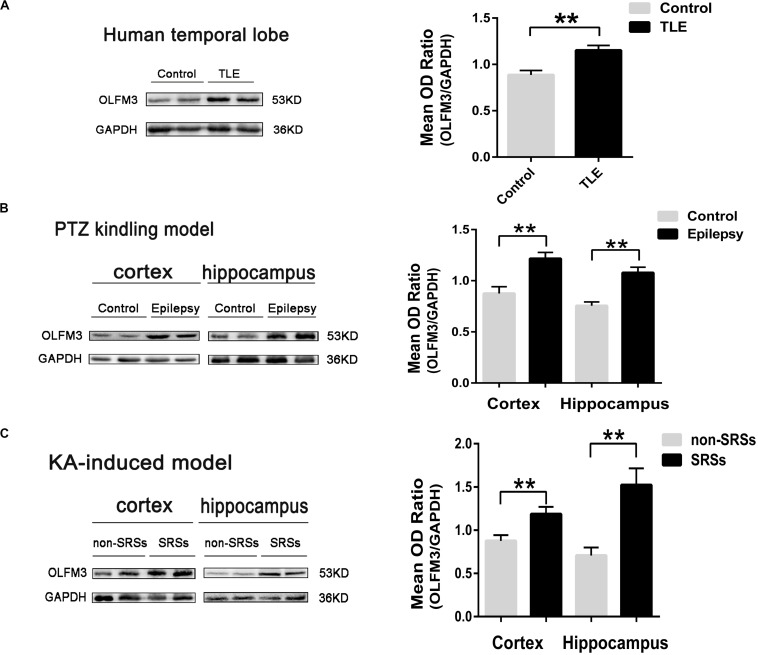
Increased OLFM3 expression in patients with TLE and the two mouse models. **(A)** Representative Western blots showing significantly increased OLFM3 expression in the cortex of patients with TLE (*n* = 24) compared with controls (*n* = 12) (**P < 0.01). **(B)** OLFM3 expression levels were significantly increased in the cortex and hippocampus of PTZ-kindled epileptic mice compared with control mice (*n* = 5 per group; **P < 0.01). **(C)** OLFM3 expression was significantly higher in the cortex and hippocampus of the spontaneous seizure group induced by KA than the non-spontaneous seizure group (*n* = 5 per group; **P < 0.01). All expression levels of OLFM3 were normalized by calculating the OD ratio of OLFM3 to GAPDH (OLFM3/GAPDH).

The detailed clinical features of TLE patients and control subjects are presented in Supplementary Tables S1, S2, respectively (Supplementary Material and Supplementary Tables S1, S2). No significant difference in age or gender was found between the TLE and control groups (*P >* 0.05, Supplementary Table S2). First, Western blotting was applied to survey OLFM3 expression levels in the cortices of patients with TLE (*n* = 24) and controls (*n* = 12). The expression of OLFM3 was significantly higher in the TLE group than the control group (Figure 1A) (*P* < 0.01). Next, OLFM3 expression in mouse models of epilepsy induced by PTZ (Figure 1B) and KA (Figure 1C) was assessed by Western blotting. As in human samples, the OLFM3 expression levels were significantly upregulated in the hippocampus and cortex of the epileptic compared with the control mice (*n* = 5 per group; *P* < 0.01).

### Localization of OLFM3 in the Human Neocortex and in the Mouse Cortex and Hippocampus

**FIGURE 2:**
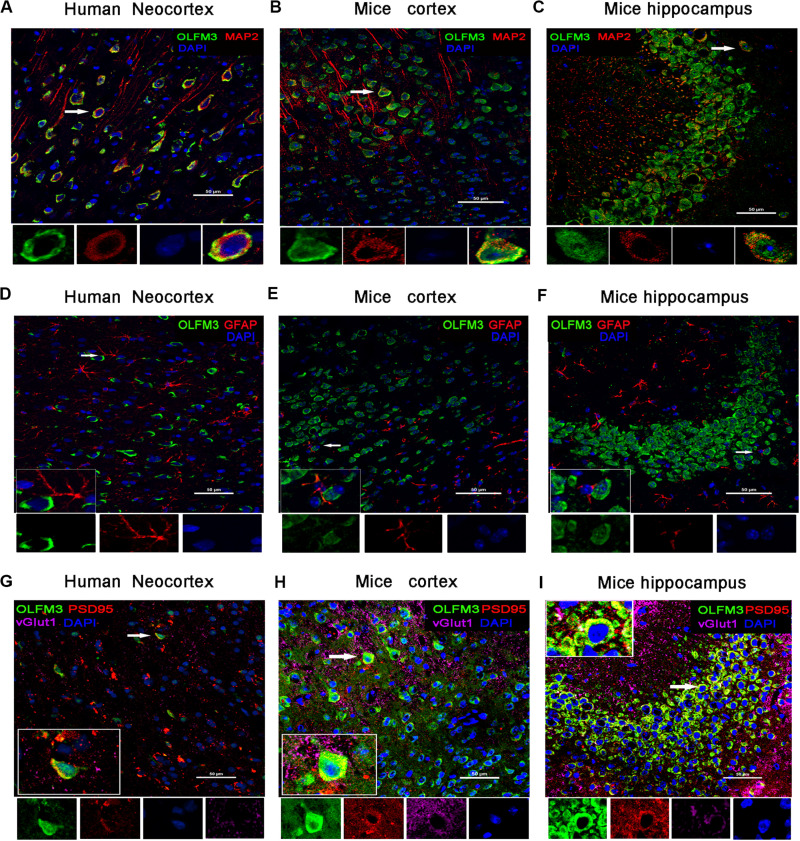
Localization of OLFM3 in the human neocortex and in the mouse cortex and hippocampus. **(A–C)** OLFM3 (green) and MAP2 (red) were co-expressed (yellow) in the human neocortex **(A)**, mouse cortex **(B)**, and mouse hippocampus **(C)**, while no co-localization of OLFM3 and GFAP (red) was detected **(D–F)**. **(G–I)** Representative images show that OLFM3 expression (green) overlapped (yellow) with that of the excitatory postsynaptic marker PSD95 (red) but not the excitatory presynaptic marker vGlut1 (purple) in the human neocortex **(G)** and mouse cortex **(H)** and hippocampus **(I)**. DAPI (blue) indicates cell nuclei. White squares indicate positive cells, and the scale bar represents 20 or 50 μm.

Immunofluorescence staining showed that OLFM3 was co-localized with the neuronal dendrite marker MAP2 (Figure 2A) but was not co-expressed with GFAP (a marker of astrocytes) in the human neocortex (Figure 2D). Furthermore, the localization of OLFM3 was examined in mice, and similar results showed that OLFM3 was co-expressed with MAP2 (Figures 2B,C) but not GFAP in both the cortex and hippocampus (Figures 2E,F). Additionally, the expression of OLFM3 overlapped with that of the excitatory postsynaptic marker PSD95 but not the excitatory presynaptic marker vGlut1 in the human neocortex and in the mouse cortex and hippocampus (Figures 2G–I).

### Effect of OLFM3-LV and OLFM3-shRNA on Epileptic Seizure Activity

**FIGURE 3:**
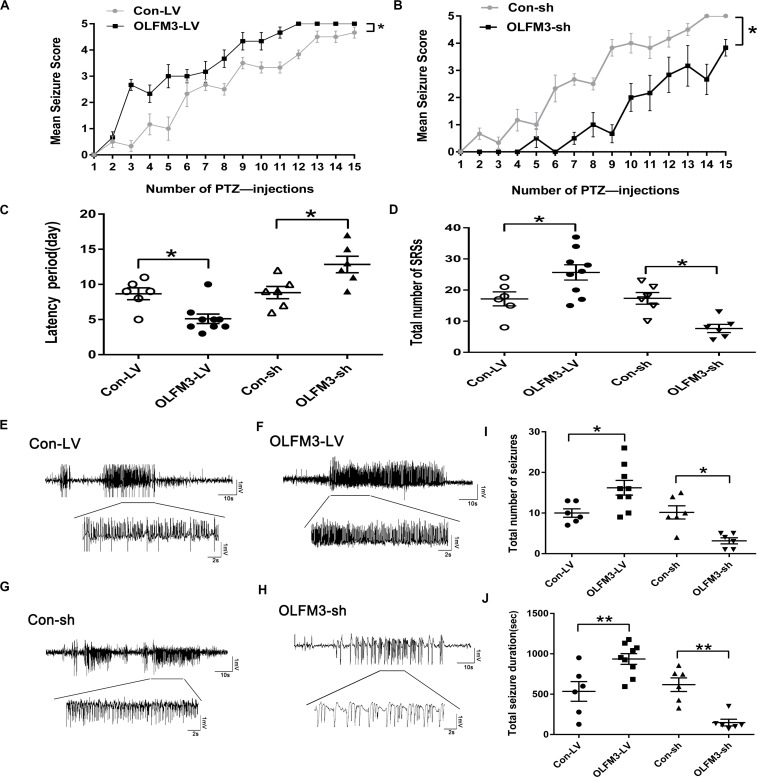
Effects of OLFM3-LV and OLFM3-shRNA on epileptic seizure activity. **(A,B)** Daily seizure scores were significantly increased in the OLFM3-LV-treated group but were significantly decreased in the OLFM3-shRNA-treated group compared with the corresponding control groups in the PTZ kindling epileptic model (*n* = 6–9 in each group) (^∗^*P* < 0.05). **(C)** The latency of SRSs in the OLFM3-LV-treated group with KA-induced epilepsy was significantly shorter than in the controls, while the latency in the OLFM3-shRNA-treated group was significantly longer than in the controls (*n* = 6–9 in each group) (^∗^*P* < 0.05). **(D)** Mice injected with OLFM3-LV had more SRSs than the control group. However, mice injected with OLFM3-shRNA had less frequent SRSs than control mice (*n* = 6–9 in each group) (^∗^*P* < 0.05). **(E–H)** Representative electrical epileptiform discharges in each group (Con-LV, OLFM3-LV, Con-sh, and OLFM3-shRNA). **(I,J)** EEG recordings showed that the mice injected with OLFM3-LV had a significantly increased frequency and total duration of electrical epileptiform discharges compared with the mice injected with Con-LV, while the frequency and total duration of the electrical epileptiform discharges were significantly decreased in the OLFM3-shRNA-treated group compared with the control group (*n* = 6–9 in each group) (^∗^*P* < 0.05, ^∗∗^*P* < 0.01).

As shown above, OLFM3 expression was increased in epileptic tissue. Follow-up experiments were performed to detect whether OLFM3 deletion might have beneficial effects on the two epilepsy models (Supplementary Material and Supplementary Figure S1). First, OLFM3 was overexpressed or knocked down locally in the hippocampus by intrahippocampal injection of a corresponding lentivirus bearing GFP. Next, GFP autofluorescence was detected in the mouse hippocampus by confocal microscopy after injection, confirming successful lentiviral infection (Supplementary Material and Supplementary Figure S2a). Moreover, Western blotting revealed that OLFM3 expression was significantly higher in the OLFM3-LV-treated than the Con-LV-treated mice but lower in the OLFM3-shRNA-treated than the Con-sh-treated mice (Supplementary Material and Supplementary Figure S2). Finally, the behavioral data for PTZ-kindled epileptic mice (*n* = 6–9 per group) (*P* < 0.05) (Figures 3A,B) and mice with KA-induced epilepsy (*n* = 6–9 per group) (*P* < 0.05) (Figures 3C,D) were analyzed. Seizure scores after each PTZ injection according to Racine’s standard scale were significantly increased in the OLFM3-LV-treated group (Figure 3A) but decreased in the OLFM3-shRNA-treated group compared with the scores in the corresponding control groups (Figure 3B). In the KA-induced epilepsy model, OLFM3-LV injection shortened the spontaneous seizure onset latency, while OLFM3-shRNA injection prolonged the onset latency compared with Con-sh injection (Figure 3C). Moreover, the OLFM3-LV-treated group had a significantly higher while the OLFM3-shRNA-treated group had a significantly lower SRS frequency than the corresponding control groups (Figure 3D). Additionally, multichannel EEG recordings were conducted *in vivo* concurrently after behavior monitoring (Figures 3E–H). The EEG recording data demonstrated that the total number and duration of the electrical epileptiform discharges were significantly increased in the OLFM3-LV-treated group but decreased in the OLFM3-shRNA-treated group compared with the corresponding control groups (*n* = 6–9 per group) (*P* < 0.05 or 0.01) (Figures 3I,J).

### Effects of OLFM3-LV and OLFM3-shRNA on Electrophysiology

**FIGURE 4:**
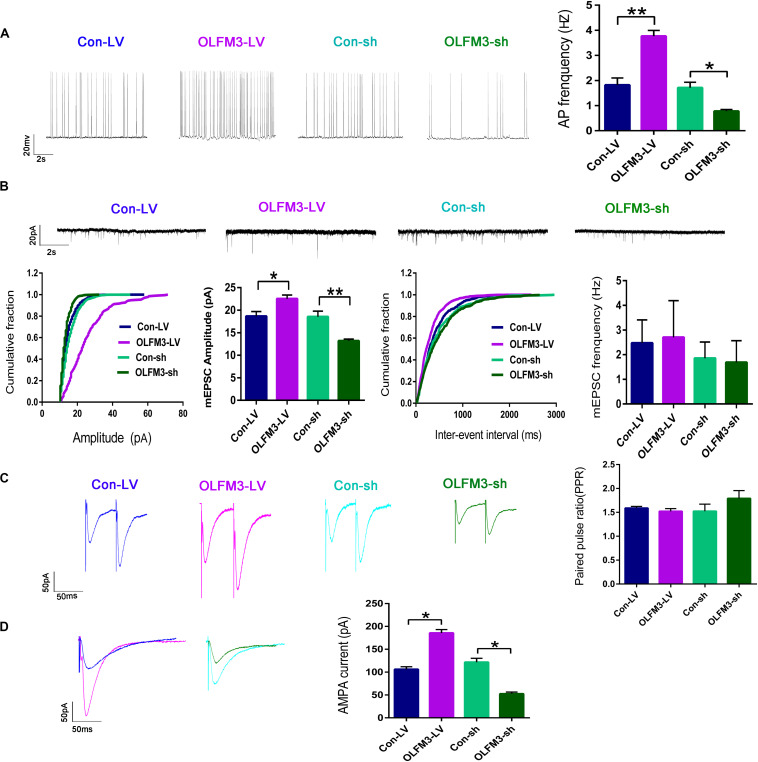
Effects of OLFM3-LV and OLFM3-shRNA on electrophysiology. **(A)** The frequency of APs was significantly higher in the OLFM3-LV group than the Con-LV group, while the frequency of APs was lower in the OLFM3-shRNA group than the Con-sh group. **(B)** The amplitude of mEPSCs was remarkably increased in the OLFM3-LV group but significantly decreased in the OLFM3-shRNA group. There was no significant difference in mEPSC frequency between the OLFM3-LV and OLFM3-shRNA groups and the corresponding control groups. **(C)** There were no significant differences in PPR between the OLFM3-LV or OLFM3-shRNA group and their corresponding control groups. **(D)** The AMPAR current amplitude was significantly increased in the OLFM3-LV group and significantly decreased in the OLFM3-shRNA group compared with the current amplitude in the corresponding control group (*n* = 5 per group; *P < 0.05, **P < 0.01).

Whole-cell patch-clamp recording was conducted to further investigate the effect of OLFM3-LV and OLFM3-shRNA on electrophysiology in an Mg^2+^-free-induced brain-slice model of epileptiform activity 14 days after OLFM3-LV or OLFM3-shRNA injection. The recording data from the pyramidal neurons in the CA1 region showed that the frequency of APs was significantly increased in the OLFM3-LV-treated group compared with the Con-LV-treated group. In contrast, the frequency of APs was significantly decreased in the OLFM3-shRNA-treated group compared with the Con-sh-treated group (*n* = 5 per group; *P* < 0.05 or 0.01) (Figure 4A). To examine whether OLFM3-LV and OLFM3-shRNA altered excitatory neurotransmission, we recorded mEPSCs. The mEPSC amplitude was significantly higher in the OLFM3-LV-treated group but lower in the OLFM3-shRNA-treated group than in the corresponding control groups (*n* = 5 per group; *P* < 0.05 or 0.01) (Figure 4B). However, there was no significant difference in the frequency of mEPSCs among the groups (*n* = 5 per group; *P* > 0.05) (Figure 4B). Furthermore, PPR was assessed to determine whether OLFM3-LV and OLFM3-shRNA altered excitatory presynaptic release. There were no significant differences in PPR between the OLFM3-LV or OLFM3-shRNA group and their corresponding controls (*n* = 5 per group; *P* > 0.05) (Figure 4C). Finally, evoked AMPAR-mediated currents were recorded to determine whether the change in excitatory neurotransmission was mediated by AMPAR currents. The results revealed that AMPAR-mediated currents were significantly increased in the OLFM3-LV group and decreased in the OLFM3-shRNA group compared with the corresponding control groups (*n* = 5 per group; *P* < 0.05) (Figure 4D).

### Effects of OLFM3-LV and OLFM3-shRNA on AMPARs

**FIGURE 5:**
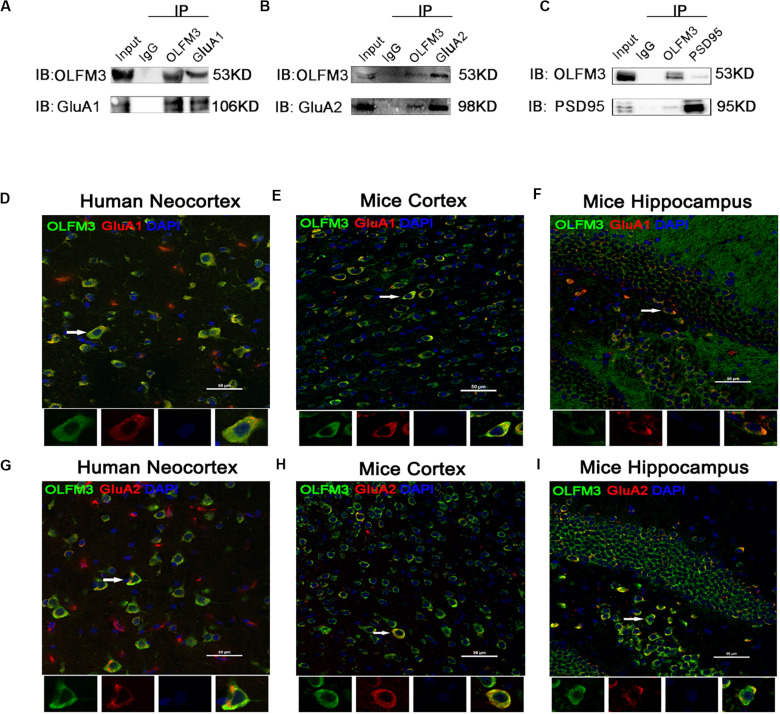
Co-localization of OLFM3 and AMPARs. **(A–C)** Co-immunoprecipitation of GluA1, GluA2, and PSD95 with OLFM3 demonstrated by the reciprocal co-immunoprecipitation of anti-OLFM3 with anti-GluA1, anti-GluA2, and anti-PSD95, respectively. Immunofluorescence revealed that the distribution of OLFM3 (green) overlapped (yellow) with that of GluA1 (red) in the human neocortex **(D)** and mouse cortex **(E)** and hippocampus **(F)**. **(G–I)** Representative immunofluorescence images show that OLFM3 (green) was co-expressed (yellow) with GluA2 (red) in the human neocortex **(G)** and mouse cortex **(H)** and hippocampus **(I)**. Cell nuclei were counterstained with DAPI (blue). The arrows and white squares indicate positive cells, and the scale bar represents 50 μm.

**FIGURE 6:**
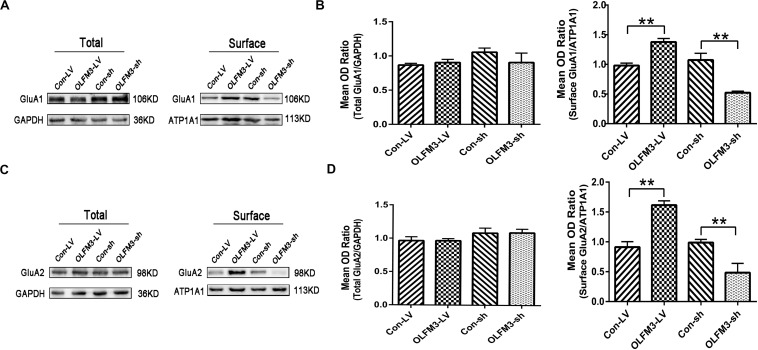
Effects of OLFM3-LV and OLFM3-shRNA on AMPARs in epileptic mice. **(A–D)** There was no significant difference in the expression of total GluA1 **(A,B)** or GluA2 **(C,D)** in the hippocampus of either the OLFM3-LV-treated or the OLFM3-shRNA-treated group compared with the corresponding control groups. However, the membrane expression levels of GluA1 **(A,B)** and GluA2 **(C,D)** were significantly higher in the OLFM3-LV group than in control mice, and the membrane expression levels of GluA1 **(A,B)** and GluA2 **(C,D)** membrane proteins were significantly lower in the OLFM3-shRNA group than the corresponding controls. Data are presented as the mean ± SEM (*n* = 6–10 in each group) (**P < 0.01).

As demonstrated above, OLFM3 was localized to the excitatory presynaptic PSD95 and increased AMPAR-mediated currents. A series of follow-up experiments were performed to survey whether OLFM3 could interact with AMPARs and alter their expression to increase AMPA currents. Using the epileptic hippocampal tissue for co-immunoprecipitation, we demonstrated that anti-OLFM3 co-immunoprecipitation with anti-GluA1, anti-GluA2 and anti-PSD95 (Figures 5A–C) but not anti-GluA3 or anti-GluA4 (Supplementary Figure S3). Furthermore, immunofluorescence showed that the expression of OLFM3 overlapped with that of GluA1 (Figures 5D–F) and GluA2 (Figures 5G–I) not only in the human neocortex but also in the mouse cortex and hippocampus. Taken together, these results verified that OLFM3 interacted with AMPARs (GluA1 and GluA2). Next, Western blotting was conducted to examine whether GluA1 and GluA2 protein expression levels in the hippocampus of mice with KA-induced epilepsy were altered by overexpression or knockdown of OLFM3; however, neither GluA1 nor GluA2 total protein levels were significantly different between the experimental and control groups (*n* = 6–10 in each group) (*P* > 0.05) (Figures 6A–D). Next, the membrane/surface expression levels of GluA1 and GluA2 were measured. The results showed that the membrane/surface expression levels of both GluA1 and GluA2 were significantly higher in the OLFM3-LV-treated group than the Con-LV-treated group, while they were significantly lower in the OLFM3-shRNA-treated group than the Con-sh-treated group (*n* = 6–10 in each group) (*P* < 0.01) (Figures 6A–D). These results revealed that OLFM3 might affect AMPA currents via interaction with AMPARs and abnormal membrane expression of the GluA1 and GluA2 proteins.

## Discussion

In this paper, we found that OLFM3 expression was significantly increased in the brains of TLE patients and two classic models (PTZ- and KA-induced mouse models of epilepsy). We present the novel finding that lentivirus-mediated overexpression of OLFM3 in the hippocampus increased the susceptibility of mice to seizures in the models. Furthermore, OLFM3 affected AMPAR currents in a brain-slice model of epileptiform activity induced by Mg2+-free medium. Most notably, we found that OLFM3 may interact with GluA1 and GluA2 to modulate seizure activity.

As a member of the olfactomidin domain family, OLFM3 has been found to increase the activity of mucin, stimulate cells to adhere to each other, promote close connections between cells, enable cell migration, and regulate the formation of the cytoskeleton during the process of cell migration (Anholt, 2014). Differences in the gene coding for OLFM3 have been found between patients with epilepsy and controls (Heinzen et al., 2010). In the present study, most of the OLFM3-positive cells in both human and mouse brain tissues co-expressed neuronal markers but not glial cell markers. The expression of OLFM3 was significantly increased in the epileptic focus specimens of patients and in the hippocampus and cortex of the classic models of epilepsy. It is not known whether the increase of OLFM3 caused by epilepsy or epilepsy caused by the increase of OLFM3. In order to explore this problem, we need to carry out follow-up experiments. However, due to the limitations of ethics, we couldn’t find the answer in human hippocampus.

Therefore, we conducted behavioral experiments on two classic epilepsy models to further explore this phenomenon. Lentivirus-mediated overexpression of OLFM3 in the hippocampus is found increased the susceptibility of mice to PTZ-induced seizures. In order to prevent false positives of a single model, we used KA-induced chronic epilepsy model for further verification. The EEG recording data from the KA-induced epileptic mice is consistent with the results of the PTZ-induced seizures.

Furthermore, whole-cell patch-clamp recordings revealed that overexpression or knockdown of OLFM3 increased or decreased AP frequency, mEPSC amplitude and AMPAR-mediated currents, respectively, in a brain-slice model of epileptiform activity induced by Mg^2+^-free medium. Previous studies have demonstrated that AMPARs are tetramers composed of GluA1-4 subunits, of which GluA1-GluA2 and GluA2-GluA3 are mainly found in mature hippocampal excitatory synapses, and the role of GluA1-GluA2 is particularly important (Mayer, 2016; Gupta, 2018; Liu et al., 2018). AMPARs mediate the fast, transient transmission of excitatory signals in the central nervous system by combining with PSD95, which anchors AMPARs to the postsynaptic membrane, connects AMPARs to other signaling molecules, and allows for the interaction with other PSD95 molecules and AMPARs modification through phosphorylation, palm acylation and so on. Interestingly, OLFM3 co-localized with the excitatory postsynaptic marker PSD95 and with GluA1 and GluA2 in this study. By mediating excitatory synaptic transmission and enhancing the excitatory postsynaptic current, AMPARs ultimately participate in the onset of epilepsy and development (Jaremko et al., 2017; Widagdo et al., 2017; Joshi et al., 2018). Thus it can be seen AMPAR complexes play a major role in the pathogenesis and development of epilepsy (Mayer, 2016; Adotevi and Leitch, 2017; Cai et al., 2017). Proteomics and mass spectrometry analysis had been revealed that OLFM3 may be involved in the formation of AMPAR complexes (Schwenk et al., 2012; Shanks et al., 2012). Additionally, Sultana et al. (2014) found that the formation of AMPAR complexes was inhibited by knocking out OLFM2, thereby reducing AMPAR-mediated learning, cognitive and motor functions in mice. Whether OLFM3 also plays a certain regulatory role in the formation of AMPAR complex to participate in the development of epilepsy, unfortunately, there has been no relevant report before.

Previous studies have shown that the transport of AMPARs to the membrane is a dynamic circulation process. After the receptor is internalized, a part is degraded by lysosomes, and the other part is returned to the membrane through cell phagocytosis after the reorganization of the cytoplasmic membrane network structure to restore its role in excitatory synaptic transmission. Changes in the amount of AMPAR in the membrane can cause changes in the excitability of the central nervous system (Derkach et al., 2007; Widagdo et al., 2016). Recently, some researchers have found that the dentate gyrus granule cells of TLE and other animal models of epilepsy form abnormal mossy fiber synapses, which indicates that the recruitment of AMPAR (especially GluA1 and GluA2 subunits) is induced (Joshi et al., 2017; Joshi and Kapur, 2018). Another study showed that GluA1 and GluA2 mediated increased excitatory transmission of hippocampal CA1 pyramidal neurons. This increase may play an important role in exacerbating menstrual seizures (Joshi et al., 2018). Other studies have further confirmed that enhanced surface expression of GluA1 in SE can induce AMPAR-mediated increase in excitatory neurotransmission in CA1 pyramidal neurons (Joshi et al., 2017). It was found that the degradation of AMPAR is regulated by the GluA2 subunit and its related proteins (Parkinson et al., 2018). The co-immunoprecipitation experiments in this study showed that OLFM3 interacted with GluA1, GluA2 and PSD95, respectively, in this study. The overexpression and knockdown of OLFM3 had no effect on the total protein of GluA1 or GluA2, but the overexpression of OLFM3 increased the membrane expression of GluA1 and GluA2 in the hippocampus, and after the decrease of OLFM3 expression, the membrane expression of GluA1 and GluA2 decreased. Therefore, OLFM3 may not participate in the synthesis or degradation of AMPAR. By regulating the translocation of GluA1 and GluA2 membrane expression, it may be related to the change in the excitability of epilepsy.

## Conclusion

In this study, we demonstrated that OLFM3 might participate in the formation of AMPAR complexes. Upregulation of OLFM3 increased the excitatory postsynaptic currents and enhanced seizure activity by increasing the surface expression of GluA1 and GluA2 in neuronal cells. Downregulation of OLFM3 may have a therapeutic effect on epilepsy. However, the exact mechanism of these effects requires further study.

## Data Availability Statement

All datasets presented in this study are included in the article/Supplementary Material.

## Ethics Statement

This study was approved by the Ethics Committee of Zunyi Medical College and Chongqing Medical University according to the World Medical Association’s Declaration of Helsinki. Before undergoing surgery, all patients or their lineal relatives provided written informed consent for use of the resected tissue in research. No potentially identifiable human images or data are presented in this study. The methods were conducted in compliance with the ethical guidelines for medical and health research involving human subjects as established by the National Institutes of Health and the Committee on Human Research at Zunyi Medical College and Chongqing Medical University. All animal experiments were performed according to the principles outlined in the National Institutes of Health guide (NIH Publication No. 8623, revised 1987). All animal experimental protocols were reviewed and approved by the Ethics Committee of Zunyi Medical College and Chongqing Medical University (Approval No. 0002648). In addition, all efforts were made to reduce the number of animals used in the experiment and to minimize their suffering.

## Author Contributions

ZX, TW, and ST conceived and designed the experiments. YG, DL, YZ, YL, and ST performed the experiments. PX, JZ, and ZL collected the data and carried out the statistical analysis. ST, CY, and XZ wrote the manuscript. All authors contributed to the preparation of the manuscript and approved the final contributions.

## Conflict of Interest

The authors declare that the research was conducted in the absence of any commercial or financial relationships that could be construed as a potential conflict of interest.

## Abbreviations

AEDs: antiepileptic drugs
AMPAR: α -amino-3-hydroxy-5-methyl-4-isoxazole-propionic acid receptor
APs: action potentials
DAPI: 4’,6-diamidino-2-phenylindole dihydrochloride
ECL: Enhanced chemiluminescence
EEGs: electroencephalograms
GFAP: glial fibrillary acidic protein
GFP: green fluorescent protein
IP: immunoprecipitation
KA: kainic acid
MAP2: microtubule-associated protein 2
mEPSCs: miniature excitatory postsynaptic currents
NMDAR: N-methyl -D-aspartate receptor
OD: optical density
OLFM3: Olfactomedin-3
PPR: paired pulse ratio
PSD-95: postsynaptic density 95
PTZ: pentylenetetrazol
PVDF: polyvinylidene fluoride
SE: status epilepticus
SPF: specific-pathogen-free
SRSs: spontaneous recurrent seizures
TLE: temporal lobe epilepsy
vGlut1: vesicular glutamate transporter 1.
