# Supplementary material for: Olfactomedin-3 Enhances Seizure Activity by Interacting With AMPA Receptors in Epilepsy Models
Source: Front Cell Dev Biol. 2020 Aug 11;8:722. doi: 10.3389/fcell.2020.00722 (PMC7431667; doi:10.3389/fcell.2020.00722)
Supplement: Supplementary file 1 [file Table_1.docx]

Supplementary material

**Supplementary results**

**
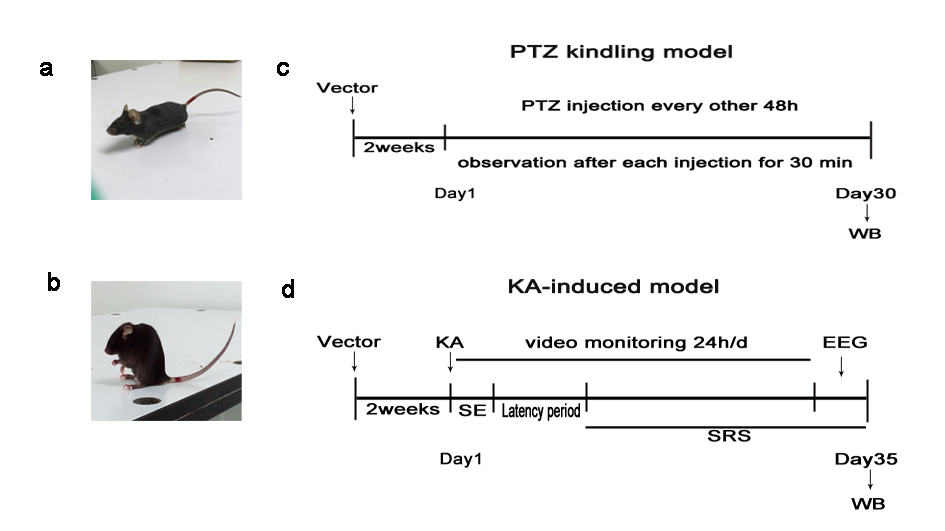
**

**Fig s1 Effects of OLFM3-LV and OLFM3-shRNA on epileptic mice. a** a mouse of the control group **b**  A mouse having a Racine stage 4 seizure, manifested as bilateral front limb twitching and an upright trunk. The process used and the behavioral observation data for PTZ kindling epileptic mice model **(c)** and KA-induced epilepsy model **(d)**


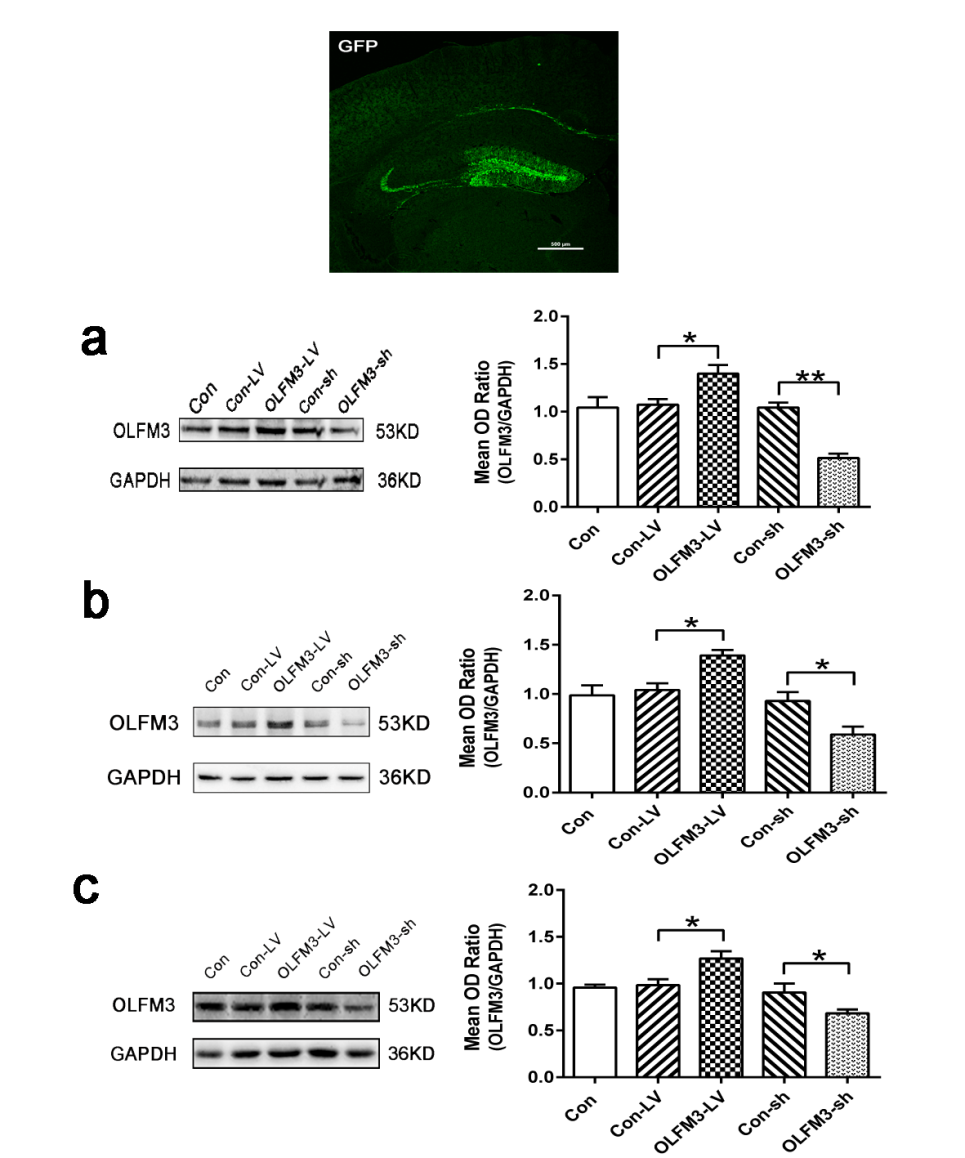


**Fig s2 Expression of GFP and OLFM3 after injection of lentiviral vectors. a** GFP was visualized in the mouse hippocampus on day 14 by confocal microscopy following lentiviral infection; scale bars represent 200 μm. Western blots indicated that OLFM3 expression was significantly increased in the OLFM3-LV-treated group compared to the Con-LV-treated group but was significantly decreased in the OLFM3-shRNA-treated group compared with the Con-shRNA-treated group.  **b and c** The expression of GFP and OLFM3 after injection of lentiviral vectors 30 and 45 day respectively. (n = 5 per group) (**P* < 0.05, ***P* < 0.01).


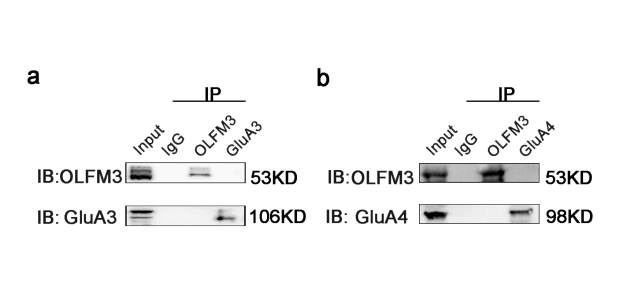


**Fig s3**  Interaction of OLFM3 with GluA3 and GluA4 in mice. There was no interaction between OLFM3 and GluA3 (a) or GluA4 (b) in mice demonstrated by co-immunoprecipitation.

**Table S1** Clinical features of TLE patients.

| Patients  No. | Gender (M/F) | Age  (y) | Course (y) | AEDs before surgery | Pathology | Resection tissue |
| --- | --- | --- | --- | --- | --- | --- |
| 1 | M | 18 | 5 | CBZ, VPA, PHT | NL | TNl |
| 2 | F | 26 | 8 | LTG, CBZ, OXC, PB | Gliosis | TNr |
| 3 | M | 35 | 10 | OXC, PB, TPM, LTG | NL,Gliosis | TNl |
| 4 | M | 32 | 7 | CBZ, TPM, PHT, VPA | NL | TNr |
| 5 | F | 29 | 5 | LTG, CBZ, PB | Gliosis | TNl |
| 6 | M | 43 | 14 | OXC, CBZ, VPA, TPM | NL | TNl |
| 7 | F | 22 | 4 | LTG, PB, CBZ, PHT | NL | TNl |
| 8 | F | 36 | 11 | LTG, CBZ, PHT, TPM | Gliosis | TNr |
| 9 | M | 25 | 5 | CBZ, VPA, PB, PHT | NL | TNl |
| 10 | F | 38 | 13 | CBZ, PB, TPM, LEV | NL | TNr |
| 11 | F | 45 | 18 | OXC, LEV, PHT, LTG | Gliosis | TNl |
| 12 | M | 24 | 7 | VPA, CBZ, LTG, LEV | NL | TNr |
| 13 | M | 21 | 4 | OXC, VPA, CBZ, PHT | NL | TNr |
| 14 | M | 37 | 15 | LTG, TPM, VPA, LEV | NL,Gliosis | TNr |
| 15 | F | 48 | 17 | CBZ, PHT, PB, LTG | NL | TNl |
| 16 | M | 40 | 12 | LEV, LTG, PB, OXC | Gliosis | TNl |
| 17 | M | 32 | 16 | LEV, CBZ, VPA, PHT | NL,Gliosis | TNl |
| 18 | F | 38 | 10 | OXC, LTG, TPM, PHT | NL | TNr |
| 19 | M | 53 | 25 | TPM, OXC, VPA, CBZ, PHT | NL,Gliosis | TNr |
| 20 | F | 28 | 9 | LEV, PB, TPM | NL | TNl |
| 21 | F | 45 | 17 | PHT, LEV, TPM, LTG | NL | TNr |
| 22 | M | 33 | 10 | VPA, OXC, TPM, LEV | Gliosis | TNr |
| 23 | F | 45 | 19 | OXC, PB, CBZ, LEV | NL | TNl |
| 24 | M | 36 | 11 | VPA, PHT, TPM, CBZ | Gliosis | TNr |

M, male; F, female; y, year(s); AEDs, antiepileptic drugs; CBZ, carbamazepine; LEV, levetiracetam; LTG, lamotrigine; OXC, oxcarbazepine; PB, phenobarbital; PHT, phenytoin; TPM, Topamax; VPA, valproic acid; TN, temporal neocortex; l, left; r, right; NL, neuron loss.

**Table S2**  Clinical features of control individuals with brain trauma.

| Patients No. | Gender (M/F) | Age  (y) | Etiology diagnosis | Resection tissue | Pathology |
| --- | --- | --- | --- | --- | --- |
| 1 | M | 23 | Trauma | TNr | N |
| 2 | F | 36 | Trauma | TNl | N |
| 3 | M | 32 | Trauma | TNr | N |
| 4 | M | 27 | Trauma | TNl | N |
| 5 | M | 40 | Trauma | TNl | N |
| 6 | F | 38 | Trauma | TNl | N |
| 7 | M | 29 | Trauma | TNr | N |
| 8 | F | 30 | Trauma | TNr | N |
| 9 | M | 50 | Trauma | TNl | N |
| 10 | F | 25 | Trauma | TNr | N |
| 11 | F | 28 | Trauma | TNr | N |
| 12 | F | 37 | Trauma | TNl | N |

F, female; M, male; y, year; TN, temporal neocortex; l, left; r, right; N, normal.
